# Production of structurally diverse sphingolipids by anaerobic marine bacteria in the euxinic Black Sea water column

**DOI:** 10.1093/ismejo/wrae153

**Published:** 2024-08-08

**Authors:** Su Ding, F A Bastiaan von Meijenfeldt, Nicole J Bale, Jaap S Sinninghe Damsté, Laura Villanueva

**Affiliations:** Department of Marine Microbiology and Biogeochemistry, NIOZ Royal Netherlands Institute for Sea Research, 1797 SZ 't Horntje, Texel, The Netherlands; Department of Marine Microbiology and Biogeochemistry, NIOZ Royal Netherlands Institute for Sea Research, 1797 SZ 't Horntje, Texel, The Netherlands; Department of Marine Microbiology and Biogeochemistry, NIOZ Royal Netherlands Institute for Sea Research, 1797 SZ 't Horntje, Texel, The Netherlands; Department of Marine Microbiology and Biogeochemistry, NIOZ Royal Netherlands Institute for Sea Research, 1797 SZ 't Horntje, Texel, The Netherlands; Department of Earth Sciences, Faculty of Geosciences, Utrecht University, 3584 CS Utrecht, The Netherlands; Department of Marine Microbiology and Biogeochemistry, NIOZ Royal Netherlands Institute for Sea Research, 1797 SZ 't Horntje, Texel, The Netherlands; Department of Biology, Faculty of Sciences, Utrecht University, 3584 CS Utrecht, The Netherlands

**Keywords:** microbial sphingolipids, Black Sea, molecular network, omics, metalipidomics, metagenomics, marine microbiology, anoxic marine waters

## Abstract

Microbial lipids, used as taxonomic markers and physiological indicators, have mainly been studied through cultivation. However, this approach is limited due to the scarcity of cultures of environmental microbes, thereby restricting insights into the diversity of lipids and their ecological roles. Addressing this limitation, here we apply metalipidomics combined with metagenomics in the Black Sea, classifying and tentatively identifying 1623 lipid-like species across 18 lipid classes. We discovered over 200 novel, abundant, and structurally diverse sphingolipids in euxinic waters, including unique 1-deoxysphingolipids with long-chain fatty acids and sulfur-containing groups. Sphingolipids were thought to be rare in bacteria and their molecular and ecological functions in bacterial membranes remain elusive. However, genomic analysis focused on sphingolipid biosynthesis genes revealed that members of 38 bacterial phyla in the Black Sea can synthesize sphingolipids, representing a 4-fold increase from previously known capabilities and accounting for up to 25% of the microbial community. These sphingolipids appear to be involved in oxidative stress response, cell wall remodeling, and are associated with the metabolism of nitrogen-containing molecules. Our findings underscore the effectiveness of multi-omics approaches in exploring microbial chemical ecology.

## Introduction

Microbial lipids are structurally diverse and have proven to be robust indicators of specific taxonomic groups [1–3]. In addition, many microbes regulate their membrane lipid composition to adapt to environmental stresses [1, 4]. For instance, certain prokaryotes modify the number of branched methyl groups or rings in their membrane lipids in response to temperature, pH, or salinity changes [5, 6]. Consequently, lipids can also be used as biomarkers for specific environmental conditions or as indicators of climate change [6–10]. The distribution of intact polar lipids has been reported in many marine settings [11–18], lakes [19], and soils [20, 21]. Recent developments in the field of “molecular omics”, employing non-targeted methodologies alongside computational techniques, have facilitated advancements in environmental metabolomics [22, 23]. This allows for a comprehensive characterization of all lipid molecules in the environment, the so-called metalipidome, aiding in the discovery of novel molecules [11, 24, 25] with unknown biological sources. Nevertheless, environmental metalipidomics studies based on non-targeted detection remain scarce [11, 25].

Traditionally, the identification of microorganisms that synthesize a specific lipid and elucidation of the lipid’s function have relied on microbial cultivation and further lab experiments. However, the lack of cultured representatives for the vast majority of microbes hinders a comprehensive understanding of the structural diversity of lipids and their molecular and ecological functions across the tree of life [26]. A different approach involves identifying lipids directly in the environment and inferring their biological sources and potential functions. Currently, most studies that suggest potential lipid producers in the environment rely on the co-variation of lipid abundance with molecular taxonomic markers, such as the 16S rRNA gene [27]. However, this approach can lead to erroneous conclusions, as not all microbes that co-vary with a molecular product necessarily have the capacity to produce it. Alternatively, it is possible to investigate the genetic capacity of a microorganism to synthesize a lipid compound of interest by targeting their lipid biosynthetic pathways [28, 29]. However, this method requires knowledge of the enzymes involved in the lipid biosynthetic pathways and sometimes depends on the direct detection by PCR amplification of the targeted protein-coding genes, which could induce biases [30, 31]. At present, metagenomic sequencing, combined with de novo assembly and genome-resolved metagenomics, provides a cultivation-independent, unbiased, and comprehensive view of the microbial community [32–34]. In principle, this method allows for the identification of likely producers of lipids, especially if biosynthesis genes are known. However, currently, non-targeted environmental metalipidomics combined with metagenomics-based lipid biosynthetic potential detection is unprecedented.

In this study, we apply a combination of environmental metalipidomics and metagenomics resulting in the identification of novel lipids and the assignment of their likely microbial sources in the water column of the Black Sea. As the lipid biosynthetic pathways leading to all microbial lipids are not fully constrained, we focus here on a group of membrane lipids, i.e. sphingolipids, for which the lipid biosynthetic pathway has been identified [35–37]. Sphingolipids play essential roles in eukaryotes as fundamental building blocks of the cell membrane and serve vital functions in cellular signaling and organization of lipid rafts, however their potential wide phylogenetic distribution within bacteria has only recently been suggested [38], and their molecular and ecological functions in bacteria remain largely unknown and are likely diverse (Supplementary Note). Our results reveal an unprecedented sphingolipid structural diversity in the water column of the Black sea, a euxinic basin harboring a high microbial diversity [17, 39, 40]. We also report largely overlooked bacterial sources of sphingolipids, with functions associated with oxidative stress, cell wall remodeling, and metabolism of small molecules in the deep anoxic waters of the Black Sea, possibly reflecting specific microbial adaptations to this euxinic environment.

## Materials and methods

### Sampling

A detailed description of sample collection, DNA and lipid extraction, and Ultra High-Pressure Liquid Chromatography (UHPLC)-high-resolution mass spectrometry (HRMS)/MS initial data processing is given in previous studies [11, 12, 27]. Briefly, suspended particulate matter (SPM) was collected in the water column of the Black Sea at 15 depths (50–2000 mbsl) during the PHOXY cruise (9–10 June 2013). The PHOX2 sampling station was located at 42°53.8’N, 30°40.7′E in the western gyre of the Black Sea. SPM was collected with McLane WTS-LV in situ pumps (McLane Laboratories Inc., Falmouth) on pre-washed 142-mm diameter 0.7-mm pore size glass fiber GF/F filters (Pall Corporation, Port Washington, NY). The filters were immediately stored at −80°C after collection.

### Lipid extraction

A modified Bligh–Dyer procedure was used to extract lipids from the freeze-dried filters [41]. Medium blanks and extraction blanks were used to subtract background compounds and contaminants, especially TAGs and phospho-DAGs, which can be commonly found in humans. The samples and blanks were extracted ultrasonically for 10 min, twice in a mixture of methanol, dichloromethane and phosphate buffer (2:1:0.8, v:v:v) and twice with a mixture of methanol, dichloromethane and aqueous trichloroacetic acid solution pH 3 (2:1:0.8, v:v:v). The organic phase was separated by adding additional dichloromethane and buffer to a final solvent ratio of 1:1:0.9 (v:v:v) and was re-extracted three times with dichloromethane and dried under a stream of N_2_ gas. The extract was redissolved in a mixture of MeOH:DCM (9:1, v:v) and filtered through 4-mm diameter 0.45-μm pore size regenerated cellulose syringe filters (Grace Alltech).

### UHPLC-HRMS/MS analysis and molecular networking

The lipid extracts were analyzed using an Agilent 1290 Infinity I UHPLC system coupled to a Q Exactive Orbitrap HRMS system (Thermo Fisher Scientific, Waltham, MA). Instrument methods are described in a previous study [12]. The raw data files generated by UHPLC-HRMS/MS were further processed using MZmine software [42]. Processing steps [43] included mass peak detection, chromatogram building, chromatogram deconvolution, isotope grouping, ion component alignment, gap filling, as well as a manual check.

The processed dataset of MS/MS spectra was further analyzed using the Feature Based Molecular Networking method [44] through the Global Natural Product Social Molecular Networking (GNPS) platform [43] to build molecular networks of the detected components. Molecular networking is a data analysis methodology utilized in untargeted metabolomics studies based on MS/MS analysis. It organizes MS/MS spectra into a network-like map, where molecules with similar spectral patterns are grouped together, indicating their structural similarity. Vector similarities were calculated by comparing pairs of spectra based on at least six matching fragment ions (peaks). This comparison takes into account the relative intensities of the fragment ions as well as the difference in precursor *m/z* values between the spectra [45]. The resulting molecular network is generated using MATLAB scripts, where each spectrum is allowed to connect to its top K scoring matches (typically up to 10 connections per node). Edges between spectra are retained if they are among the top K matches for both spectra and if the vector similarity score, represented as a cosine value, exceeds a user-defined threshold. In this study, a cosine value of 0.6 was used, with a cosine value of 1.0 indicating identical spectra. The exported molecular network was then imported into the visualization program Cytoscape [46, 47] for further analysis.

Each node in the molecular network stands for an individual molecule associated with a specific MS/MS spectrum. Due to the extraction and analytical methods, and based on annotations of the same data in a previous study [11], most of the ion components from the molecular network we detected were lipids, thus we used the term metalipidome where the ion components are discussed. Most of the molecules that clustered together in the subnetworks were either analogs of each other with an identical headgroup or with a similar core, differing by simple transformations such as alkylation, unsaturation, and glycosylation.

### Metalipidome annotation

The MS/MS spectra in the metalipidomic molecular network only resulted in <2% annotations based on a search through the GNPS library, as described in our previous work [11]. These 239 annotations included ca. 20 contaminants, and mostly TAGs, highly unsaturated PC-DAGs, PE-DAGs from plankton and algal species, leaving the vast majority of ion components unknown. Our previous work [11] further annotated the most important lipids in the molecular network (with obvious changes of abundance through the water column or as a central node linked by several other lipids in the network) either by comparison to data from previous studies [14, 16–21, 27, 41, 48–59] or putatively identified based on accurate mass and MS/MS fragmentation. We submitted the mass spectra of these annotated lipids to the GNPS library for this study’s further analysis and for public use.

In total, the mass spectra of 249 representative lipids of the 18 major lipid classes from the molecular network were submitted to the GNPS library. Based on the annotated lipids from each subnetwork, we determined major lipid classes and used them for the lipid composition analysis.

### Lipid standard calibration

Calibration of lipid peak intensity using external standards is necessary to correct for large differences in ionization response between lipid classes, especially for the lipids with different polar headgroups. Supplementary Tables 1 and 2 detail the information on standards used to quantify each lipid class. We assumed that all lipids from the same class should have similar response factors, thus curves based on a single representative lipid species were used to calibrate each lipid class [60]. For sphingolipids Cers and LysCers, an average response factor of ceramide mix was used for calibration (Supplementary Tables 1 and 2). 1-deoxyCer-associated sphingolipid classes were calibrated using an external standard 1-deoxyCer (d18:1/24:0) and Gly-Cers were calibrated using Glucosyl (β) C12 Cer. For the class of lipids with no standards available (e.g. OLs), a response factor from another class of amino lipids (DGTS) was used for calculation. The concentration of all the lipids of each MS/MS spectra (ng L^−1^) was then added as metadata for the metalipidome visualization across the water column of the Black Sea sample set (50–2000 mbsl).

### DNA extraction

The DNA extraction from SPMs across the water column of the Black Sea was reported in a previous study [27, 61]. In short, DNA was extracted with the DNA Power Soil isolation kit (Mo Bio Laboratories, Inc., Carlsbad, CA). The integrity and concentration of the extracted DNA were tested by agarose gel electrophoresis and NanoDrop (Thermo Scientific, Waltham, MA) quantification.

### Metagenomic sequencing

The metagenomes of the samples used in this study were sequenced before [61]. We sequenced the metagenomes again here, much deeper this time. Previous metagenomic sequencing in ref. [61] generated 44 887 523 reads after quality control, whereas here we generated 4 266 171 462 reads after quality control—an almost 100-fold increase in sequencing depth. TruSeq nano libraries were sequenced with Illumina MiSeq at Utrecht Sequencing Facility generating 251 base pair paired-end reads. All bioinformatic tools used to analyze these metagenomes were run with default parameters unless otherwise stated. FastQC v0.11.9 (https://www.bioinformatics.babraham.ac.uk/projects/fastqc/) was used for quality control, and quality and adapter trimming was performed with Trimmomatic v0.39 [62] with options “ILLUMINACLIP:TruSeq3-PE-2.fa:2:30:12:2:TRUE MAXINFO:40:0.6 MINLEN:40”. After trimming, the samples contained 197 509 556–412 252 887 paired-end sequencing reads.

### Assembly and binning

The 15 samples were individually assembled with metaSPAdes v3.15.1 [63] and assembly quality was assessed with QUAST v5.0.2 [64]. Sequencing reads were mapped back to the assembled scaffolds with BWA-MEM v2.2.1 [65] generating 15 × 15 mappings. Depth files were generated with the jgi_summarize_bam_contig_depths scripts that comes with MetaBAT2 [66]. The scaffolds were binned per sample with MetaBAT2 v2.2.15 [66], CONCOCT v1.1.0 [67], and MaxBin2 v2.2.7 [68]. For CONCOCT, the scaffolds were cut in chunks of 10 000 base pairs, and scaffolds shorter than 2500 base pairs were excluded from binning. For MaxBin2, scaffolds shorter than 2500 base pairs were excluded from binning and the 40 universal marker genes set was used. Metagenome-assembled genomes (MAGs) generated by MetaBAT2, CONCOCT, and MaxBin2 were used as input for DAS Tool v1.1.3 [69] with the BLAST search engine to arrive at a final MAG set per sample. MAG quality was assessed with CheckM v1.1.3 [70] in the lineage-specific workflow.

Taxonomy was assigned to scaffolds and MAGs with medium to high quality (> 50% completeness, < 10% contamination [71]) with Contig Annotation Tool (CAT) and Bin Annotation Tool (BAT) [72], respectively, from the CAT pack software suite v5.2.3. A reference database was constructed based on the set of non-redundant proteins in GTDB r207 [73]. Proteins were predicted with Prodigal v2.6.3 in metagenomic mode [74] and aligned to the reference database with DIAMOND v2.0.6 [75] with the –top parameter set to 11 for CAT and the –top parameter set to 6 for BAT. With BAT’s default parameters (-f 0.3), a MAG may get multiple taxonomic annotations. In those cases, the majority classification (-f 0.5) was used.

### Homology searches of ceramide biosynthesis genes and phylogeny of serine palmitoyltransferase homologs

Genes were predicted on the scaffolds with Prodigal v2.6.3 in metagenomic mode [74]. We queried the bacterial serine palmitoyltransferase (Spt), bacterial ceramide synthase (bCerS), and ceramide reductase (CerR) protein sequences from ref. [38] (query sequences and TBLASTN results from Supplementary Data 1 of ref. [38]) in the predicted protein sequences with BLASTP v2.12.0 [76], with a database size of 1 × 10^8^ to make *e*-values comparable across samples. Spt homologs with an *e*-value $\le$ 1 × 10^−70^ and query coverage $\ge$ 70% to the best hit query sequence based on *e*-value were selected for further phylogenetic tree building with known α-oxoamine synthase sequences to constrain Spt activity. Some of these hits were part of longer protein sequences. To prevent the alignment of possible non-homologous regions, we extracted the BLAST High Scoring Pair from hits with a sequence coverage <70%, and in other cases the entire predicted protein. The hits were clustered together with the Spt query sequences and 16 α-oxoamine synthases with different enzymatic activities [10] with CD-HIT v4.8.1 [77] at 90% sequence identity. The resulting 2666 representative sequences were aligned with MAFFT v7.505 using the L-INS-i algorithm, and the alignment was trimmed with trimAl v1.4.rev15 [78] in gappyout mode, leaving 411 sites. A maximum likelihood phylogenetic tree was reconstructed with IQ-TREE v2.1.2 [79] using model finder [80] with nuclear amino-acid models, and 1000 ultrafast bootstraps [81]. The LG + F + R10 model was chosen based on Bayesian Information Criterion (BIC). The tree was visualized in iTOL [82]. Homologs of bCerS and CerR were filtered based on *e*-value $\le$ 1 × 10^−30^ and query coverage$\ge$ 50%.

To identify possible eukaryotic Spt sequences, we queried the Spt sequences of *Homo sapiens* (NP_006406.1), *Arabidopsis thaliana* (NP_190447.1), and *Saccharomyces cerevisiae* (CAA56805.1) in the predicted protein sequences with BLASTP, with a database size of 1 × 10^8^, and filtered based on *e*-value $\le$ 1 × 10^−70^ and query coverage $\ge$ 70%.

### Abundance estimation

Taxonomic profiles were generated based on mapping of the sequencing reads to the scaffolds with BWA-MEM v2.2.1 [65]. Taxonomic annotation of a read was inherited from the BAT annotation of the MAG when possible or else from the CAT annotation of the assembled scaffold, similar to ref. [34]. We similarly generated profiles for the mappings that could be associated with Spt-like proteins via the MAG or unbinned scaffold.

### Pangenome-wide association analyses

The MAGs whose (completeness – 5× contamination) ≥ 70% were annotated in six functional universes provided by InterPro [83,84] (Conserved Domains Database, Gene Ontology (GO), InterProScan, NCBIfam, pathways, and Pfam) with InterProScan v5.63-95.0 [83,84]. In each SPM sample, functions that were more commonly present or absent (qualitatively) in potential sphingolipid producers compared to MAGs that lack Spt-like proteins were identified with Fisher’s exact test, using Bonferroni *P*-value correction. Functions that were present in higher or lower abundance (quantitatively) were identified with the Mann–Whitney U test, using Bonferroni *P*-value correction. We performed the tests per SPM sample to allow for the identification of niche-specific associations.

### Co-abundance analyses

Spearman’s rank correlation coefficient (ρ) between the abundance of binned scaffolds and the abundance of sphingolipids across the water column of the Black Sea were calculated with the SciPy library. For abundance of the scaffolds we took the mapping depth normalized by the number of reads after quality control per sample. Circos plots were generated with pyCirclize (https://github.com/moshi4/pyCirclize). In these plots, relative summed abundance of scaffolds or sphingolipids was based on the sum of the abundance across the water column, relative to the highest value found for any scaffold or sphingolipid, respectively. In Fig. 4, summed abundances of the individual scaffolds are depicted, and in Fig. 5 the average summed abundance of all scaffolds in a MAG.

Connections between sphingolipid and scaffolds that contain Spt-like proteins with ρ $\ge$ 0.9 were visualized in a network [85] using Cytoscape (version 3.9.0) with a Spring Embedded layout [46, 47].

To represent the abundance weight of scaffolds and sphingolipids across the water column of the Black Sea (110–2000 mbsl) in this network, a color gradient stripe ranging from orange to navy blue was employed. The abundance weight of the *i*th MS/MS and scaffolds among the samples was calculated using the following formula:


$$ {P}_i={\sum}_{j=1}^t{P}_{ij}\ast j $$

where ${P}_{ij}$ corresponds to the relative frequency of the *i*th MS/MS or scaffolds (*i* = 1, 2, $\dots$, *m*) in the *j*th sample (*j* = 1, 2, $\dots$, *t*), to illustrate the dominant distribution of a specific sphingolipid or scaffold across the water column. For instance, when ${P}_{ij}$ has a value close to 9, it represents a sphingolipid or scaffold that exhibits significant abundance in the upper euxinic zone (~110 mbsl). Such instances are denoted by the color orange on the gradient stripe. Conversely, when ${P}_{ij}$ approaches a value of 15, it indicates a predominant distribution in the deep waters (~2000 mbsl), and is consequently represented by the color dark blue on the stripe. By examining the network, a trend can be observed from the upper euxinic zone to the deep sea in terms of abundance levels.

## Results and discussion

The Black Sea is the largest stratified anoxic marine basin in the world, holding promise for the discovery of novel biological molecules due to the distinct microbial niches caused by its redox zonation. To comprehensively characterize its complete metalipidome and its potential contributors, we collected SPM covering the full range of redox zones from 50 to 2000 m below sea level (mbsl; 15 depths): the oxic zone (50–70 mbsl), and the anoxic zone (80–2000 mbsl), which is further subdivided in the suboxic zone (80–105 mbsl; no detectable sulfide), and the euxinic zone (110–2000 mbsl; sulfidic) [27,86]. Prior investigations [27] of the physicochemical characteristics of the Black Sea water column (see Supplementary Fig. 1) revealed that oxygen concentrations exhibited a noticeable decline from the surface layer downward, decreasing from ~78 μM at 50 mbsl to levels under the detection limit around 80 mbsl. The same trend was also found for nitrate, which had a concentration of ~1.3 μM at 50 mbsl and concentrations under the detection limit at ~90 mbsl. Sulfide was not detected in the surface layer and appeared at ~110 mbsl (0.85 μM), which marks the upper boundary of the euxinic zone (here defined as sulfide concentration > 0.2 μM). Lipids and DNA were extracted based on previous methods [12, 27].

### Characterization of the metalipidome of the Black Sea

**Figure 1:**
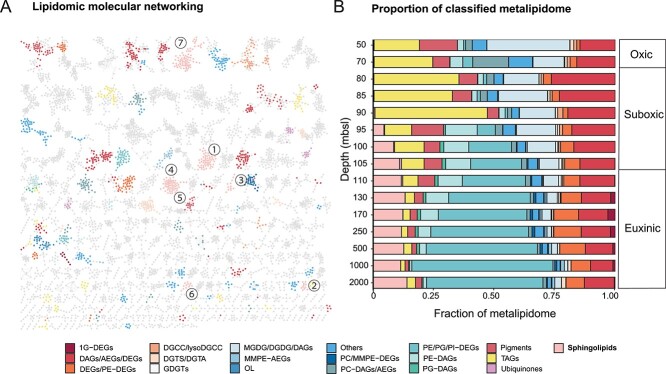
Metalipidome composition of the Black Sea. (A) Molecular network of the metalipidome of the water column (combining all SPM samples from different water depths). Nodes represent MS/MS mass spectra of ion components within an analytical window of *m/z* 350–2000, which are connected based on spectral similarity. Edges between lipids are retained if the structural similarity score, represented as a cosine value, exceeds a user-defined threshold. In this study, a cosine value of 0.6 was used, with a cosine value of 1.0 indicating identical spectra. Nodes with colors are major lipid classes shown in (B) and numbers refer to the seven sphingolipid classes shown in Fig. 2(D). The spatial orientation of the nodes in the MS/MS network is randomly generated by Cytoscape^42^ and does not relate to relationships between the subnetworks. (B) the relative abundance of major lipid classes in the water column of the Black Sea. Relative abundances of lipids were calibrated using the response factor of external standards (see “experimental procedure” for details). Major lipid classes that did not have a relative abundance >1% of the classified metalipidome in multiple SPM samples are classified as “other”. Lipid abbreviations: 1G-DEGs, monoglycosyldietherglycerols; DAGs, diacylglycerols; DEGs, dietherglycerols; AEGs, acyletherglycerols; TAGs, triacylglycerol; DGTS, diacylglycerylhydroxymethyltrimethyl-(N,N,N)-homoserine; DGCC, diacylglycerylcarboxyhydroxymethylcholine; DGTA, diacylglyceryl-hydroxymethyl)-tri-methyl-b-alanine; OL, ornithine lipid; PC, phosphatidylcholine; PE, phosphatidylethanolamine; PG, phosphatidylglycerol; PI, phosphatidylinositol; MMPE, phosphatidyl-(N)-methylethanolamine; MGDG, monoglycosyldiacylglycerol; DGDG, diglycosyldiacylglycerol; GDGT, glycerol dialkyl glycerol tetraethers.

Lipid extracts obtained from the SPM across the water column of the Black Sea were analyzed with UHPLC coupled with HRMS [12]. The selected analytical window for the MS/MS mass spectra of ion components detection was *m/z* 350–2000, targeting the relatively large components of the environmental metabolome. To characterize the chemical diversity of this metalipidome, the MS/MS mass spectra of ion components were subjected to molecular networking based on their cosine similarities (Fig. 1A). A clustering of ion components in the molecular network suggests similar molecular structures, for example as analogs with an identical polar headgroup or similar core that differ through simple transformations such as alkylation, unsaturation, and glycosylation. Only a small fraction (less than 2%, 239 out of 12 031) of the components in the metalipidome could be directly annotated through reference database searches, most of which were likely contaminants or lipids such as TAGs and specific phospholipids associated with phytoplankton and algal species [11]. However, we successfully classified or putatively identified 1623 lipid species into 18 major lipid classes within the molecular network. This was done based on manual inspection of specific abundant components in the subnetworks [11] (Fig. 1B).

The 18 major classes of lipids (Fig. 1B; abbreviations in the caption of Fig. 1) included core lipids (DAGs, AEGs, and DEGs), phospholipids (PE, PG, PI, and MMPE headgroups connected to DAGs, AEGs, and DEGs), glycolipids (1G-DEGs, MGDG, DGDG), GDGTs, betaine lipids (DGCC, lysoDGCC, DGTS/DGTA), ornithine lipids (OLs), pigments, triacylglycerols (TAGs), ubiquinones, and sphingolipids. The lipids with the highest summed relative abundance at 50–90 mbsl depths were TAGs, constituting 19–46% of all classified lipids, followed by glycolipids (13–34%), and DAGs (15–26%) (Fig. 1B).

**Figure 2:**
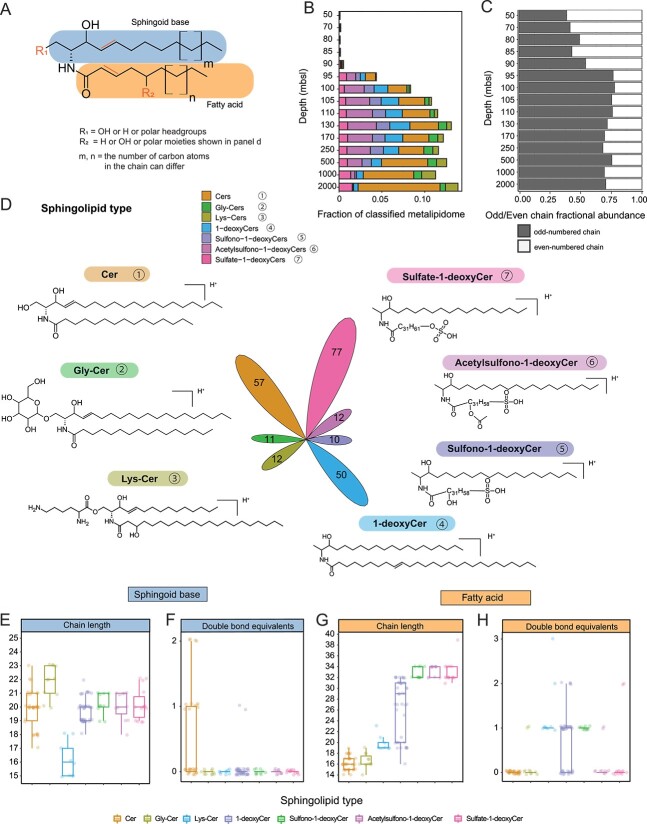
Sphingolipids in the Black Sea water column. (A) General structure of sphingolipids consisting of a sphingoid base linked to a fatty acid via an amide-bond. The number of carbon atoms of the sphingoid base and fatty acid moieties ranged from 15 to 22 and 14 to 34, respectively. (B) The relative abundance of seven sphingolipid subclasses identified in the metalipidome. (C) The variation in the fractional abundance of odd- and even-numbered chain sphingolipids with depth in the water column of the Black Sea. An “odd-numbered chain” sphingolipid contains either an odd chain sphingoid base and/or an odd chain fatty acid. An “even-numbered chain” sphingolipid contains even chains of both the sphingoid base and fatty acid. (D) Structures of each representative sphingolipid subclass. The flower plot area with the numbers in the center represents the number of distinct ion components within each subclass. The ranges of (E) chain length and (F) the number of DBEs of the sphingoid base for the various sphingolipid classes. The ranges of (G) chain length and (H) the DBEs of the fatty acid base. Out of the 229 identified sphingolipids, we were able to assign molecular structures with confident information on chain-length carbon atoms and DBEs to 122 (see source dataset). These 122 sphingolipids were subjected to detailed Fig. 2(E–H) analysis. In (A), for the class of Cer, R_1_ = OH, R_2_ = H; for the class of Gly-Cer, R_1_ = Gly, R_2_ = H; for the class of Lys-Cer, R_1_ = Lys, R_2_ = OH; for the class of 1-deoxyCer, R_1_ = H, R_2_ = H; for the class of Sulfono-1-deoxyCer, R_1_ = H, R_2_ = SO_3_H and OH (the position of these two groups cannot be determined based on the mass spectra and they are not connected to each other); for the class of Acetylsulfono-1-deoxyCer, R_1_ = H, R_2_ = SO_3_H and CH_3_COO (the position of these two groups cannot be determined based on the mass spectra and they are not connected to each other); for the class of Sulfate-1-deoxyCer, R1 = H, R2 = SO_4_H.

TAGs are energy storage molecules of eukaryotic algae such as *Euglenophyceae*, *Cryptophyceae*, and *Eustigmatophyceae* [87], and their relative abundance falls below 5% in the euxinic zone (Fig. 1b). Unexpectedly, sphingolipids reached an unprecedented high abundance of up to 14% of the classified metalipidome in the deep euxinic waters (Fig. 1b). Sphingolipids are composed of a sphingosine backbone linked to a fatty acid via an amide bond (Fig. 2). Even though sphingolipids in marine environments were previously thought to be primarily produced by eukaryotic algae and their associated viruses [88–90], they accounted for <0.5% in the oxic and upper suboxic zone (50–90 mbsl), the primary niches for eukaryotes in the Black Sea. In contrast, sphingolipids had a mean relative abundance of 12% in the euxinic zone, making them the third most abundant lipid class in these deep waters, after phospholipids (mainly PE/PG/PI-DEGs; 8–58%) and core lipids (mainly AEGs and DEGs; 9–18%), suggesting a possible prokaryotic source. However, unlike phospholipids, sphingolipids are thought to be rare in bacteria, although, recently, their presence has been inferred in nine bacterial phyla [38] (further information about bacterial sphingolipids can be found in the Supplementary Note). A previous study identified a single subclass of sphingolipids in the Black Sea, glycosylceramides (Gly-Cers), and suggested that they were produced by (facultative) anaerobic bacteria because of the high abundance of these lipids in the anoxic deep waters [16]. Before delving into the potential microbial origins of the sphingolipids in the deep waters of the Black Sea, we first describe their structural diversity.

### Structural diversity and distribution of sphingolipids

The metalipidomic approach revealed that the Black Sea contained 229 distinct structures of sphingolipids in the deeper suboxic waters (Fig. 2). These sphingolipid structures varied in polar headgroups/moieties, chain length, and degree of unsaturation. The 229 distinct sphingolipids structures can be divided into seven subclasses: ceramides (Cers; also including saturated ceramides), Gly-Cers, lysine-ceramides (Lys-Cers), 1-deoxyceramides (1-deoxyCers), sulfono-1-deoxyceramides (sulfono-1-deoxyCers), Acetylsulfono-1-deoxyceramides (Acsulfono-1-deoxyCers), and sulfate-1-deoxyceramides (sulfate-1-deoxyCers) (Fig. 2D). We also examined the core lipid composition of each subclass of sphingolipids, focusing on the number of carbon atoms and double bond equivalents (DBEs) in their sphingoid base and their fatty acid moiety (Fig. 2 and Supplementary Fig. 2), as these two parameters represent a large fraction of the structural diversity within the molecular subnetworks.

The Cers subnetwork comprised 57 different structures, and it represented the most abundant sphingolipid subclass in the euxinic zone, accounting for 3–10% of the classified metalipidome. Approximately half of the Cer structures identified (median 46%) exhibited one DBE in their sphingoid base, indicating that the remaining Cers were dihydroCers (Fig. 2C). Gly-Cers had their highest summed relative abundance at 130–1000 mbsl, and the core lipid composition of Gly-Cers was largely consistent with previously identified sphingolipids [16,18,91], with sphingoid bases ranging from 19–23 carbon atoms and C_14_ - C_19_ acyl moieties (Fig. 2E, G). Lys-Cers have not been reported before in natural environments, and had their highest summed relative abundance in the deepest waters. The novel Lys-Cers had the shortest base sphingoid chain length (median C_17_; Fig. 2E).

The four groups of 1-deoxyCers contained 149 structures, including many that have not been previously reported in the natural environment nor in microbial cultures (see below). Together they contributed 2–8% of classified lipids in the euxinic zone. 1-deoxyCers lack the C1-hydroxyl group of the classical ceramides on their sphingoid backbone. Due to the lack of the hydroxy moiety, these uncommon “headless” sphingolipids were hypothesized to be unable to form complex sphingolipids [92,93] (see Gly-Cers and Lys-Cers in Fig. 2D and the polar functional groups at their C1-OH position). Our identification of the other three 1-deoxyCer subclasses shows, however, that they are in fact capable of bearing polar moieties, such as sulfate, sulfono, and acetylsulfono on their fatty acid chains. The relative abundance of all the 1-deoxyCers-associated sphingolipids increased to a maximum at 130 mbsl and decreased again with depth, except for sulfate-1-deoxyCers, which had their highest summed relative abundance in the deepest waters. The four subclasses of 1-deoxyCer-associated sphingolipids exhibited sphingoid bases that were similar to each other, with 18–22 carbon atoms and mostly no unsaturations (Fig. 2E, F). In contrast, 1-deoxyCers contained the widest range in fatty acid chain lengths of all sphingolipid subclasses with 16–32 carbon atoms, whereas the three subclasses with additional polar moieties had similar fatty acid chain lengths consisting mostly of 32–34 carbon atoms (Fig. 2G). The longest fatty acid chain observed in the Black Sea was a C_39_ from a Sulfate-1-deoxyCer. 1-deoxyCers had between 0 and 2 DBEs on their fatty acid chains, all Sulfono-1-deoxyCers possessed 1 fatty acid chain DBE, whereas acsulfono-1-deoxyCers and sulfate-1-deoxyCers predominantly lacked fatty acid chain DBEs.

The sphingoid bases of 1-deoxyCers (1-deoxysphinganines) were initially discovered in the marine bivalve *Mactromeris polynyma* [94], and have been associated with other eukaryotes such as fungi, other bivalves, sponges, and mammals [92,93,95,96]. The very long (i.e. > 31) fatty acid chains of the 1-deoxyCer-associated sphingolipids in the Black Sea have to our knowledge not been reported in any living organism. Sphingolipids containing “long-chain fatty acids” (LCFAs) of eukaryotic origin with a chain length of 22 carbon atoms have been found in mammals [97–102], plants [103–107], invertebrates [108], and fungi [37,106,109,110]. These eukaryotic LCFAs consistently exhibit an even number of carbon atoms (Supplementary Fig. 3). In marine environments, the eukaryotic alga *Emiliania huxleyi* and its associated viruses have been observed to synthesize Gly-Cers containing LCFAs with 22 carbon atoms [88,89,111]. The 1-deoxyCer-associated sphingolipids of the Black Sea contain fatty acids with chain lengths up to 39 carbon atoms. Over 50% of these LCFAs possessed an odd chain length (Fig. 2C and Supplementary Fig. 3). The absence of odd-chain LCFAs in known sphingolipids of eukaryotes, and the observation that odd-chain fatty acids in intact polar lipids occur predominantly in bacteria [20,112], moreover may suggests that the sphingolipids in the deep waters of the Black Sea are produced by bacteria.

### The Black Sea harbors a diverse bacterial community of sphingolipid producers

To comprehensively identify likely microbial producers of the unusual sphingolipids in the Black Sea, we deeply sequenced the metagenomes of the SPM that was used for lipid analysis, followed by de novo assembly and binning of the assembled scaffolds in MAGs.

**Figure 3:**
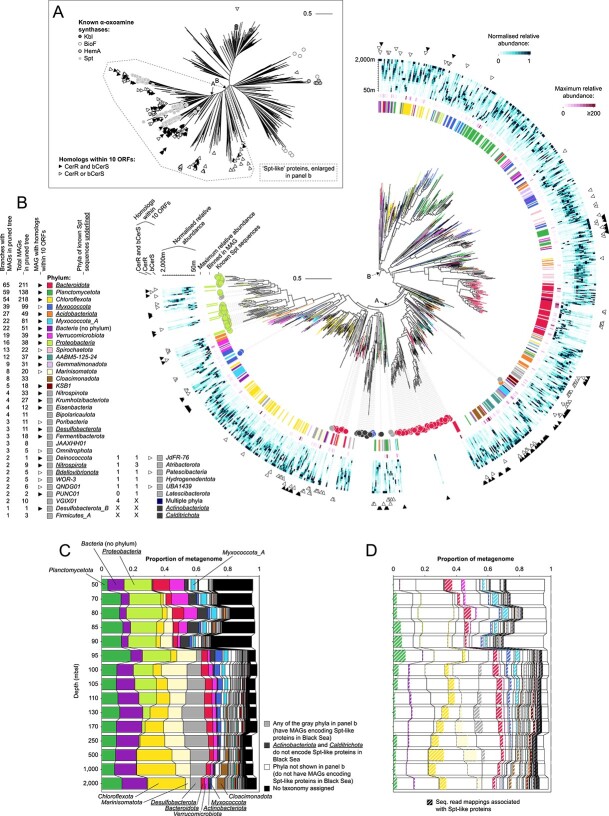
Potential sphingolipid producers in the Black Sea based on the presence of Spt-like proteins encoded on metagenomes. (A) Phylogenetic tree of Black Sea Spt homologs and reference sequences. The tree contains 2666 representative protein sequences that represent 8524 hits from the Black Sea, 273 known Spt sequences, and 16 known homologous sequences with different enzymatic activities (the α-oxoamine synthases Kbl, BioF, and HemA). Co-localization of genes encoding CerR and bCers with Spt homologs on the scaffold is indicated with triangles. The clades containing all known Spt sequences (clade A) and most sequences with CerR and bCerS encoded in close proximity on the scaffold (clade B, which includes clade A) both have high bootstrap support (≥ 98). We call all proteins from clade B “Spt-like” proteins. Spt homologs that fall outside clade B are likely α-oxoamine synthases with divergent enzymatic activity not involved in sphingolipid biosynthesis. An annotated tree with bootstrap support can be found in Supplementary Data 2. (B) Zoom-in on clade B. Taxonomic annotation at the rank phylum of medium- to high-quality MAGs (> 50% completeness, < 10% contamination) in which Spt-like proteins are binned (1271 in total) is indicated with color-coding of the branches and around the tree, Spt-like proteins encoded on unbinned scaffolds are not colored. Phyla that are represented by <5 branches are color-coded gray. Taxonomic affiliation of known Spt sequences is indicated in circles around the tree. Scale bars in (A) and (B) show the mean number of substitutions per site. (C) Community profile based on sequencing read mapping to scaffolds. Taxonomic annotation of the MAG is used if the scaffold is binned, and of the scaffold otherwise. Stacked bars add up to less than 1 because not all reads can be mapped to scaffolds. Phyla are sorted according to summed relative abundance. (d) Idem to (C) but with the fraction of sequencing read mappings colored that are associated with an Spt-like protein via the MAG or unbinned scaffold. Spt, serine palmitoyltransferase; bCerS, bacterial ceramide synthase; CerR, ceramide reductase; ORF, open reading frame; n., number; MAG, metagenome assembled genome; rel., relative; tax., taxonomy; seq., sequencing. Source data for the figure can be found in Supplementary Data 2 and 3. The figure is created with BioRender.com.

Next, we queried the predicted protein sequences on the ~1.64 × 10^8^ assembled scaffolds for the key Cer biosynthesis enzyme Spt. Spt catalyzes the condensation of L-serine and palmitoyl-coenzyme A as the first step in the biosynthesis of the ceramide backbone, and can alternatively use alanine as a substrate instead of serine in the biosynthesis of the 1-deoxyCer backbone (Supplementary Fig. 4) [38, 113–116]. We hypothesize that Spt can also use other amino acids as a substrate, like lysine to generate Lys-Cer. Spt is part of the α-oxoamine synthases protein family that contains diverse enzymatic activities [10] and even with our stringent search criteria (*e*-value $\le$ 1 × 10^−70^, query coverage $\ge$ 70%) not all identified homologs of the queried Spt proteins (“Spt homologs”; Supplementary Data 1) may have Spt enzymatic activity. To identify the homologs with likely Spt activity, we aligned the 8524 Black Sea hits and a set of known α-oxoamine synthases (Supplementary Data 2, Supplementary Fig. 5) and constructed a phylogenetic tree (Fig. 3a; Supplementary Data 2), hypothesizing that phylogenetic clustering with known Spt sequences implies conserved enzymatic activity. We also queried the predicted protein sequences for bCerS and CerR, which, together with Spt, constitute the known bacterial (1-deoxy)Cer backbone biosynthesis pathway (Supplementary Fig. 4) and are often found clustered on the genome [38,116]. By using these search criteria for sphingolipid biosynthesis genes, we aim to more accurately pinpoint potential producers based on their genomic capability.

The phylogenetic tree of Spt homologs contained a clade that included all previously known Spt sequences (Fig. 3A, B, clade A) and a larger clade that in addition to this known Spt diversity included more distantly-related proteins that also had CerR and bCerS homologs encoded in close proximity on the same scaffold (Fig. 3A, B, clade B). We considered this larger clade to potentially code for an Spt-like enzymatic activity, and the microorganisms that encode these proteins (called “Spt-like” proteins from hereon) to have biosynthetic potential to produce sphingolipids. Spt homologs that fall outside this clade are likely α-oxoamine synthases with divergent enzymatic activity not involved in sphingolipid biosynthesis.

Our phylogenetic approach to identifying the enzymatic activity of Spt homologs showed many cases in which the gene encoding the Spt-like protein was not co-localized on the genome with genes encoding bCerS and CerR. For example, none of the 33 *Cloacimonadota* MAGs had bCerS or CerR encoded within a distance of 10 open reading frames of the gene encoding the Spt-like protein (Fig. 3B), possibly indicating rearrangement of the sphingolipid biosynthesis genomic cluster, or replacement of the enzymatic activity of bCerS and CerR by alternative non-homologous proteins. It has previously been observed that the three genes are also not encoded in close proximity on the genomes of sphingolipid-producing members of *Bacteroidota* [38], indicating that genomic co-localization is not required for sphingolipid biosynthesis. Alternatively, fragmented assembly and incomplete binning may result in an underestimation of the genomic co-localization of these genes.

The clade with Spt-like proteins encompasses a much broader phylogenetic diversity of Spt than previously identified (i.e. clade A is nested within clade B), supporting a diverse community of potential sphingolipid producers present throughout the Black Sea water column (Fig. 3B). Of the 5069 medium- to high-quality MAGs (> 50% completeness, < 10% contamination [71]) obtained, 1271 encoded Spt-like proteins as defined above. These MAGs have diverse taxonomic affiliations encompassing 38 bacterial phyla, expanding the known diversity of potential sphingolipid-producing phyla from 9 (identified in ref. [38] based on genomic presence of Spt, bCerS, and CerR homologs; see Supplementary Data 2 for the taxonomic annotation of these sequences based on the Genome Taxonomy Database [73]) to 40 (Fig. 3b). No archaeal MAGs were identified encoding Spt-like proteins, suggesting that archaea in the Black Sea do not produce sphingolipids or use a different biosynthetic pathway. Whereas we cannot exclude the possibility that some Spt-like sequences were erroneously binned and thus incorrectly associated with a phylum, the high number of MAGs encoding Spt-like proteins for many phyla (e.g. 138 *Planctomycetota*, 218 *Chloroflexota*, 22 *Verrucomicrobiota*, 33 *Cloacimonadota*; Fig. 3B) suggest that this is unlikely to influence our observation that the Black Sea harbors a diverse and previously unknown community of sphingolipid producers.

### Sphingolipid producers are abundant and have distinct niches

Because we targeted all assembled scaffolds of the deeply sequenced metagenomes, our homology searches provide a comprehensive overview of the potential sphingolipid producers in the water column, with only microorganisms with low relative abundance, whose DNA was not sequenced or assembled into scaffolds, overlooked. We generated taxonomic abundance profiles based on mapping of the sequencing reads to the assembled scaffolds, inheriting the taxonomic annotation of the MAG when possible or else of the assembled scaffold, an approach that has been shown to give an accurate and comprehensive view of the microbiome [34] (Fig. 3C). We similarly generated profiles for the mappings that could be associated with Spt-like proteins via the MAG or unbinned scaffold (Fig. 3D). This approach revealed a high abundance of potential sphingolipid producers based on the presence of the Spt-like proteins throughout the water column, with a minimum of 7% of the total sequenced DNA of the microbial community in the oxic zone and upper suboxic zone attributable to potential sphingolipid producers, and up to 25% in the euxinic zone.

Even though we found potential sphingolipid producers in many phyla, in most cases not all of their members had the potential to synthesize sphingolipids. For example, the 38 *Proteobacteria* MAGs that are potential sphingolipid producers (Fig. 3A) represented only a minority of total sequenced *Proteobacteria* DNA (Fig. 3D). In contrast, a large fraction of *Bacteroidota* sequenced DNA which was abundant throughout the water column and of *Myxococcota_A* sequenced DNA which was abundant in the oxic and suboxic zone was attributed to potential sphingolipid producers (Fig. 3D). The abundance profiles of other phyla showed niche-based differentiation of individual members. For example, even though *Planctomycetota* were abundant throughout the water column, potential sphingolipid-producing members of the phylum peaked in the redoxcline. Potential sphingolipid-producing members of *Desulfobacterota* peaked in the upper euxinic zone, and of *Chloroflexota* and *Marinosomatota* in the deep waters (Fig. 3D). These microbial abundance patterns likely represent specific niches in the stratified Black Sea in which sphingolipid producers reside, as ultimately reflected in the abundance profiles of the different sphingolipid subclasses.

Finally, we addressed the possibility that (some of) the unusual sphingolipids were produced by eukaryotes. None of the scaffolds and MAGs that contained any Spt homolog, including the sequences that fell outside the selected clade of likely Spt enzymatic activity, had a eukaryotic taxonomic annotation (Supplementary Data 1). In addition, we queried the predicted protein sequences with known eukaryotic Spt sequences. This homology search revealed 26 hits that were not identified with our original bacterial Spt homology searches (Supplementary Data 1). The hits had the highest abundance in the top water layer and a low relative abundance overall (Supplementary Fig. 6C), and are thus unlikely to have significantly contributed to the observed lipid profiles in the suboxic and euxinic waters. We conclude that the sphingolipids in the Black Sea are most likely produced by the bacterial community, which showed niche-based differentiation in the stratified water column.

### Identification of potential producers of distinct sphingolipids based on co-abundance

**Figure 4:**
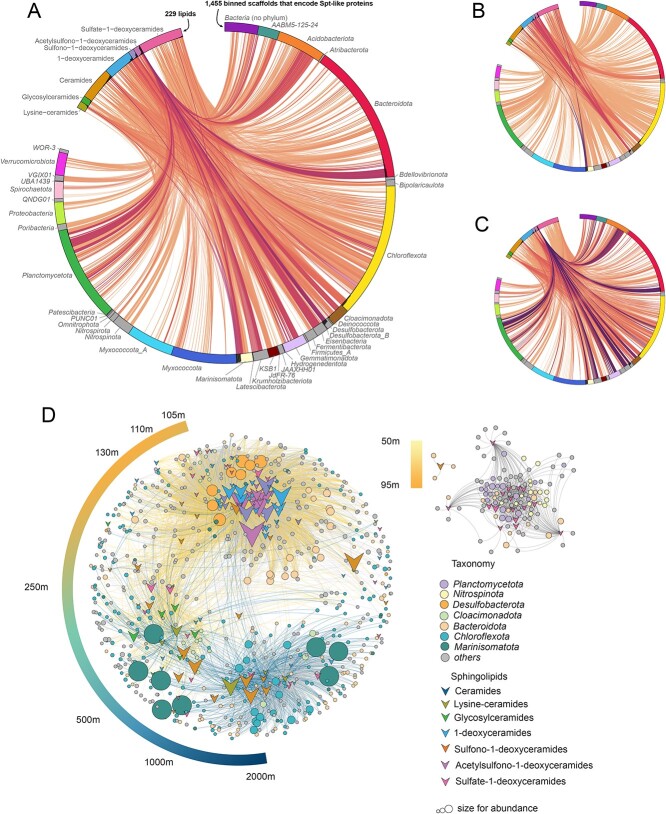
Correlations between the abundance profile of 229 sphingolipids and 1455 scaffolds that encode Spt-like proteins and that were binned in MAGs. (A) Lines depict spearman rank correlations coefficients ≥0.9 between the abundance profile of the lipid and the abundance profile of the scaffold. The line color represents the abundance of the scaffolds and of the lipids, where darker colors show connections between both high abundant lipids (summed across the water column) and high abundant scaffolds (summed across the water column). Colors scale between 0 (light; a relative summed abundance of 0 for both lipids and scaffolds) and 2 (dark; a relative summed abundance of 1 for both lipids and scaffolds). Relative summed abundance of lipids and scaffolds is shown as a black bar chart in the outer ring. (B) Idem to (A) but with the lines colored according to scaffold abundance, where darker colors show connections with scaffolds that have a high summed abundance across the water column. (C) Idem to (A) but with lines colored according to lipid abundance, where darker colors show connections with lipids that have a high summed abundance across the water column. (D) Network visualization of the same data. Edges with a ρ $\ge$ 0.9 are shown. Edge colors (see gradient scale bars) represent the sphingolipid abundance weight across the water column of the Black Sea (details can be found in Materials and methods). The abundance weight (${P}_{ij}$) is determined as a factor of the dominant distribution of a specific sphingolipid across the water column. For instance, ${P}_{ij}$ with a value close to 9 represents a sphingolipid with a dominant abundance at the upper euxinic zone (~110 mbsl), and is denoted by a light color. Conversely, when ${P}_{ij}$ approaches a value of 15, it indicates a predominant distribution in the deep waters (~2000 mbsl) and is represented by a dark color.

In order to identify potential producers of distinct sphingolipids, we correlated the abundance profiles of the 229 sphingolipids with the abundance profiles of the 1455 scaffolds that encode Spt-like proteins and that were binned in MAGs (Fig. 4). 133 sphingolipid structures had a Spearman rank correlation coefficient (ρ) ≥ 0.9 with 728 binned scaffolds and were visualized in a network (Fig. 4D). Connections between the most abundant lipids (Supplementary Fig. 6A) and most abundant MAGs (Supplementary Fig. 6B), revealed that 1-deoxyCers, sulfono-1-deoxyCers, and acsulfono-1-deoxyCers, which were dominant in the upper euxinic zone (110–250 mbsl), may be primarily produced by abundant *Desulfobacterota* and/or *Bacteroidota* (Fig. 4). *Bacteroidota* are known to synthesize sphingolipids in the mammalian gastrointestinal tract which modulate the host’s immune response [115], where they have been observed to synthesize 1-deoxyCers. *Desulfobacterota* is a phylum of sulfate-reducing bacteria and had the highest abundance of any microorganism that encodes Spt-like proteins 100–130 mbsl (Supplementary Fig. 6B).

Cers, Gly-Cers, and Lys-Cers had their highest abundance in the deep waters (500–2000 mbsl). These lipid subclasses showed strong correlations with *Marinisomatota* and *Chloroflexota* (of the taxonomic class *Anaerolineae*), which occurred abundantly at these depths (Fig. 4, Supplementary Fig. 6B). *Marinisomatota* is a highly diverse bacterial phylum predicted to be involved in sulfur and nitrogen cycles [26,117,118], and had the highest abundance of any microorganism that encoded an Spt-like protein across the water column, with a maximum at 500 mbsl. *Anaerolineae* have been found in marine sediments as chemoheterotrophs, where they may use sulfur, iron, or manganese as electron sources for growth and survival [119–121]. Both *Marinisomatota* and *Chloroflexota* have not previously been identified as potential sphingolipid producers. Finally, sulfate-1-deoxyCers showed connections with a variety of bacteria spanning the upper euxinic zone to the bottom waters (Fig. 4), including the aforementioned phyla, suggesting that they may be produced by multiple anaerobic bacterial species in multiple niches.

**Figure 5:**
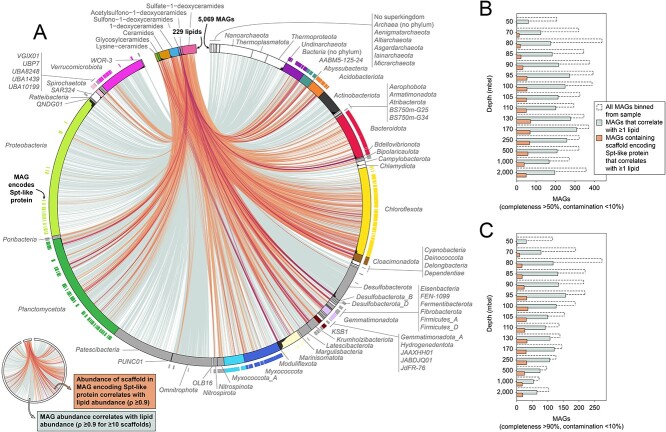
Correlations between the abundance profiles of 229 sphingolipids and MAGs irrespective of genomic presence of Spt-like proteins. (A) Gray lines depict MAG-lipid pairs in which the abundance profile of at least 10 scaffolds in the MAG have a spearman rank correlation coefficient (ρ) ≥0.9 with the abundance profile of the lipid, but in which the MAG does not encode an Spt-like protein or in which the abundance profile of the scaffold on which the Spt-like protein is encoded does not have a ρ ≥0.9 with the lipid. These cases thus likely represent false positives. A minimum of 10 scaffolds was chosen to reduce the influence of noise on the correlations, as a MAG may contain many scaffolds and their abundance profiles can slightly differ. Orange/red lines depict MAG-lipid pairs in which the abundance profile of the scaffold on which the Spt-like protein is encoded has a ρ ≥0.9 with the lipid. The line color represents the abundance of the scaffolds and of the lipids, where darker colors show connections between both high abundant lipids (summed across the water column) and high abundant scaffolds (summed across the water column). Colors scale between 0 (light; a relative summed relative abundance of 0 for both lipids and scaffolds) and 2 (dark; a relative summed relative abundance of 1 for both lipids and scaffolds). Relative summed abundance of lipids and scaffolds is shown as a black bar chart in the outer ring. (B) MAGs with a completeness >50% divided according to the sample from which they were binned. A MAG is colored gray if it correlates with at least one sphingolipid according to the criteria in (A), and orange if the scaffold that encode its Spt-like protein has a ρ ≥0.9 with at least one sphingolipid. (C) Idem as (B) but only including MAGs with a completeness >90%.

Previous studies identifying potential producers of lipids detected in the environment, correlated lipid abundances with the abundance of microbial groups based on 16S rRNA gene amplicon sequencing or metagenomics-based taxonomic profiling [27,122–124]. This approach may however lead to false positives due to correlations unrelated to causation, for example because microorganisms reside in similar niches as the actual lipid producer and thus have comparable abundance profiles. The approach used here, in which only the microorganisms that have the biosynthetic capacity to construct a lipid are correlated with the lipid’s abundance, provides a meaningful selection criterion to eliminate false positives. In order to quantify the improvement of this targeted co-abundance approach over the traditional approach of correlating lipids with all identified microorganisms, we performed another correlation analysis between the abundance profiles of the 229 sphingolipid ion components and all 5069 medium- to high-quality MAGs, irrespective of whether they had the genomic capacity to synthesize sphingolipids (Fig. 5). Out of the 1 160 801 connections (229 lipids × 5069 MAGs), we found that 56 227 had a ρ ≥ 0.9 and would be considered putative lipid-producer pairs based on co-abundance alone (Fig. 5), even though many of the MAGs do not encode Spt-like proteins and thus likely do not have the genomic capacity to produce any of the sphingolipids (Fig. 5A). MAGs with a ρ ≥ 0.9 to at least one sphingolipid belong to 62 different phyla. In contrast, when correlations were based on the presence of Spt-like proteins within a MAG, only 8825 connections had a ρ ≥ 0.9 (Fig. 5A), and only 38 phyla encode Spt-like proteins as discussed above. The six-fold reduction in putative lipid-producer pairs when including the genomic capacity to make a lipid highlights the magnitude of the problem of false positives in co-abundance studies.

Whereas we cannot exclude the possibility that some of the MAGs (completeness >50%) in which no Spt-like proteins were found do encode the protein but were incompletely binned (i.e. the protein is missing from the MAG due to methodological reasons) and thus represent false negatives, the picture is qualitatively similar when looking at the subset of MAGs with a higher >90% completeness (Fig. 5B versus Fig. 5C). It is also worth highlighting correlations with archaeal groups that have never been reported to synthesize sphingolipids (Fig. 5A), including Nanoarchaeota with streamlined genomes that have very limited lipid biosynthetic capacities [58]. We conclude that the large offset between correlations with any of the MAGs and correlations of the MAGs that encode Spt-like proteins is mainly driven by false positives, and shows the risk of determining biological sources of a molecule on the basis of correlation with only taxonomic abundance profiles.

### Search for the potential roles of sphingolipids in the Black Sea

To assess the potential role of sphingolipids in the environment, we used the 5069 medium- to high-quality MAGs to identify genomic functions that were positively or negatively associated with potential sphingolipid producers throughout the 15 depths in the water column (Materials and methods). Positively associated (enriched) functions may be involved in sphingolipid biosynthesis, or be related to an ecological role of sphingolipids in the environment. Negatively associated (depleted) functions may point to a function that the sphingolipids replace. We focused on functions that were shared by a large fraction of the phyla that were potential sphingolipid producers as these functions are candidates for a general role of sphingolipids within the environment, as opposed to taxon-specific associations.

**Figure 6:**
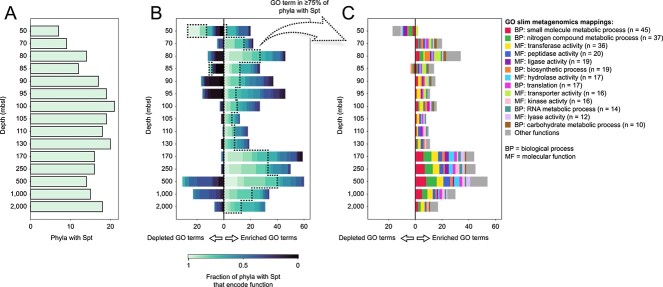
Pangenome-wide association analysis identifies functions that are depleted and enriched in MAGs that contain Spt-like proteins. (A) Number of phyla represented by the 704 MAGs that contain Spt-like proteins and whose (completeness—5× contamination) ≥ 70%. (B) Number of GO terms that are depleted (to the left of 0) or enriched (to the right of 0) in MAGs containing Spt-like proteins compared to MAGs that do not contain Spt-like proteins based on the (qualitative) Fisher’s exact test. The test compares all MAGs that were binned from a specific depth in the water column. GO terms are colored according to the fraction of phyla with Spt-like proteins at that depth that contain at least one member that encodes the function. For the same figure for five other functional universes as well identification of depleted and enriched functions based on the (quantitative) Mann–Whitney U test, see Supplementary Fig. 7. (C) Mapping of GO terms in (A) that are present in at least 75% of phyla that encode Spt-like proteins to GO slim metagenomics. GO terms can have multiple GO slim mappings. All depleted and enriched GO terms and their GO slim metagenomics mappings can be found in Supplementary Data 4.

More functions were enriched than depleted in potential sphingolipids producers throughout the water column, with few depleted functions overall (Fig. 6, Supplementary Fig. 7, Supplementary Data 4). This lack of negative associations suggests that the biosynthesis of sphingolipids does not consistently replace other functions in the Black Sea, and reflects the diverse roles of sphingolipids within bacteria that may differ between phyla or even between strains. Enriched functions that were present in at least 75% of the sphingolipid producing phyla differed with depth (Fig. 6B, C), with transporter associations prevalent in the top part of the water column and associations with small molecule and nitrogen compound metabolic processes and transferases in the euxinic zone (Fig. 6C). Many enriched functions were related to enzymatic activity (Fig. 6C), possibly suggesting a link between sphingolipids and microbial metabolism. Certain gene families with unknown functions were enriched (Supplementary Data 4), which shows a lack of understanding of sphingolipid biosynthesis and/or their producers in the Black Sea. Specific enriched functions were related to the metabolism of nitrogen-containing molecules (i.e. amino acids, nucleotides), oxidative stress response (an association also observed in plants [125]), and cell wall remodeling (Supplementary Data 4), possibly reflecting the role of sphingolipids either in the recycling of small molecules, and/or in adaptation to the euxinic zone of the Black Sea. These results provide a first and intriguing look into the role of sphingolipids in the Black Sea specifically and potentially in other environmental settings, pending future confirmation in laboratory cultures.

## Conclusions

Recent advances in meta-omics technologies, including metagenomics, metatranscriptomics, metaproteomics, metabolomics, and metalipidomics, have revolutionized our capacity to characterize microbial communities in various environments [23, 126, 127]. However, to date, there have been few studies that have effectively combined metagenomics with metalipidomics to elucidate the microbial taxa and their metabolites in complex settings [22, 23]. In an analysis of global microbial community samples collected for the Earth Microbiome Project [23], it was demonstrated that metabolite diversity exhibits turnover and nestedness, related to both microbial communities and the environment. In our study, we present the combined application of integrated metalipidomics and metagenomics. This approach has been pivotal in studying a diverse array of lipids, leading to the identification of previously unknown sphingolipids and the prediction of their potential producers and functions in complex environmental matrices, and showing the potential of using this methodological approach in other environments. Further studies are needed to understand whether the novel sphingolipids detected here are taxonomically or environmentally specific, and their role in the cell in environmental settings.

## Supplementary Material

Supplementary_Material_wrae153

## Data Availability

The processed data (.mgf and .csv) with the molecular network and detailed parameter settings can be accessed at the GNPS platform: https://gnps.ucsd.edu/ProteoSAFe/status.jsp?task=2e274c97c13e4f7d8e38637ffa9f0bee. 249 representative lipids of the 18 major lipid classes from the water column of the Black Sea were submitted to the GNPS library: https://gnps.ucsd.edu/ProteoSAFe/status.jsp?task=dc888b40c9c848fc9ece1738e9e434f0. The source data of figures are available at Zenodo at DOI: 10.5281/zenodo.11453757. The metagenomes and assemblies are available at the European Nucleotide Archive under project accession PRJEB76940, the MAGs and taxonomic annotation of all scaffolds at Zenodo at DOI: 10.5281/zenodo.11453757.

## Code availability

All code used in this study is available at Zenodo at DOI: 10.5281/zenodo.11453757.
